# High-risk human papillomavirus infection in HIV-positive African women living in Europe

**DOI:** 10.7448/IAS.16.1.18023

**Published:** 2013-02-14

**Authors:** Deborah Konopnicki, Yannick Manigart, Christine Gilles, Patricia Barlow, Jérome de Marchin, Francesco Feoli, Denis Larsimont, Marc Delforge, Stéphane De Wit, Nathan Clumeck

**Affiliations:** 1 Department of Infectious Diseases, Saint-Pierre University Hospital, Brussels Free University, Brussels, Belgium; 2 Department of Gynaecology, Saint-Pierre University Hospital, Brussels Free University, Brussels, Belgium; 3 Molecular Biology Laboratory, Institut Jules Bordet, Brussels Free University, Brussels, Belgium; 4 Department of Pathology, Institut Jules Bordet, Brussels Free University, Brussels, Belgium

**Keywords:** HPV, HIV, prevalence, incidence, women, Europe, CD4 cell count, cervix

## Abstract

**Introduction:**

Cervical infection with high-risk human papillomavirus (HRHPV) induces cervical cancer and is present in 14% of women in Europe. We assessed the prevalence and incidence of cervical HRHPV in a cohort of HIV-positive women living in Belgium.

**Methods:**

Prospective observational program of screening and follow up of HRHPV cervical infection performed by Hybrid Capture in 825 HIV-positive women between 2002 and 2011. Women without normal cervix at baseline were excluded.

**Results:**

The final analysis included 652 women: median age 38 years, African origin (81%), median HIV follow-up (66 months), median CD4 count (426 cells/μL) and 79% on antiretroviral therapy (cART). At baseline, HRHPV prevalence was 43% and decreased significantly as both age and CD4 cell count increased: highest prevalence (100%) in women <30 years and <200 CD4/μL and lowest (19%) in women >40 years and >500 CD4/μL (p<0.0001, multivariate analysis). The relative risk (RR) to carry HRHPV at baseline decreases proportionally by 11% for each 5 years-age increase and by 11% for each 100 CD4 cells/μL rise (RR=0.89, 95% CI: 0.85-0.93; p<0.0001, Poisson regression for both). During follow-up, incidence rate of HRHPV was 13.4 per 100 women-years.

**Conclusions:**

We found a high HRHPV prevalence of 43% and an incidence rate of 13 per 100 women-years in this cohort of HIV-positive women living in Europe and on cART. Women under 40 years-age had the highest prevalence even with CD4 count >350 cells/μL. The magnitude of HRHPV epidemiology should prompt to evaluate the clinical efficacy of vaccines against HPV in HIV-infected women.

## Introduction

Human papillomavirus (HPV) may induce various diseases in women depending on the genotype: high-risk (HR) genotypes are associated with cervical, vulvar and anal cancer and low risk (LR) with benign condyloma [1]. It is estimated that 12% of women with normal cervical cytology are infected with HR HPV worldwide [2]. Sub-Saharan Africa has the highest prevalence with 24%, whereas Europe has 14%. Age is one of the main factors influencing these figures: prevalence peaks before 25 years, then decreases progressively until a second peak after 45 years. HIV infection is also an important associated condition: progressive immunodeficiency significantly increases the prevalence and the persistence of HR HPV infection and worsens its evolution leading more commonly to cervical high-grade squamous intraepithelial lesions (HSIL) and cervical cancer (CC) [3, 4].

Several large studies have demonstrated the clinical efficacy of two prophylactic vaccines against HPV that protect against persistent HR HPV, HSIL and CC in HIV-negative adolescent girls and women [5, 6]. A similar immunogenicity (level of antibody response) and safety profiles of the quadrivalent HPV vaccine have been recently confirmed in HIV-positive girls and women [7–9]. However, data of the clinical efficacy against HPV-induced lesions have not yet been reported despite the higher magnitude of the HPV morbidity is this population.

A better understanding of the natural history of HR HPV cervical infection and its epidemiology in HIV-positive women is essential before designing studies evaluating vaccination of these women against HPV.

Most studies performed in HIV-positive women have reported cervical HPV prevalence mixing both LR and HR HPV and few have generated incidence rates; most of them were performed in the United States or in Africa in women not receiving combined antiretroviral therapy (cART) and very few have reported data coming from European countries. To analyze the epidemiology and the natural history of cervical HR HPV in HIV-positive women treated with cART, we initiated a prospective longitudinal cohort in 2002. We report here on the prevalence and incidence of HR HPV infection in this cohort of HIV-positive women living in Belgium.

## Materials and methods

Since 2002 an ongoing prospective observational programme of screening and follow-up of HR HPV infection is systematically offered to all women followed for HIV-1 infection in our AIDS reference centre. Women are seen by a dedicated gynaecologist within the centre and are then followed every year or more often if they have complaints or abnormal smear results. At the first and subsequent visits, cytology on Pap smear (according to the Bethesda Classification) and HR HPV screen are performed on a liquid based sample with cytological brush (ThinPrep^®^ and SurePath™). HR HPV (types 16, 18, 31, 33, 35, 39, 45, 51, 52, 56, 58, 59 and 68) are detected by Hybrid Capture (hc2 High-Risk HPV DNA Test, Digene^®^, USA). In the case of abnormal cytology, women undergo a colposcopy and a cervical biopsy. Women with previous hysterectomy or conization or HSIL or CC both confirmed by biopsy were not included in the cohort as their cervix could not be considered as intact anymore and the natural history of HR HPV infection could be altered. Women who developed HSIL or CC or who had a hysterectomy after being included in the screening programme were censored at the time of the event.

A prospective database collected all results (HR HPV detection, Pap smear and biopsy) as well as surrogate markers of HIV infection: HIV acquisition risk factor, ethnical origin, AIDS stage according to the 1993 CDC classification, duration of HIV-follow up, median CD4 cell count, receiving cART or not, median duration of cART, level of viral load (VL); smoking status and previous pregnancies were also collected.

The screening programme and the data collection were both approved by the Ethics Committee of Saint-Pierre University Hospital in Brussels.

Chi-square or Fisher's exact and Kruskal–Wallis tests were performed for univariate analysis for categorical data and continuous data, respectively. Logistic regression was used for multivariate analysis. To estimate the relative risk (RR) and confidence intervals (CI) of HR HPV carriage according to age and CD4 cell count level, we used a modified Poisson regression model by using robust error variances [10].

To calculate the incidence of new HR HPV detection, we considered all women with a first negative HR HPV test followed by at least one second sample.

## Results

### Patients’ characteristics

From 2002 to February 2011, 825 women had at least one cervical HPV screening. Women with previous HSIL or CC confirmed by biopsy (*n*=141) or non-cancerous hysterectomy (NCHST) (*n*=32) were excluded, leaving 652 patients for final analysis. Women who developed HSIL or CC (*n*=29) or had NCHST (*n*=5) after being included in the screening programme were censored at the time of the event.

The cohort consisted mainly of sub-Saharan African women (*n*=528, 81%) with heterosexual HIV acquisition (*n*=613, 94%) and non-smoking habits (*n*=476, 73%).

Among the 528 women with African ethnical origin, 422 (79%) had immigrated to Belgium and we could retrieve the exact date of arrival for 385 women (91%). The median age at time of immigration was 31 years (IQ25-75: 25–37) and 64% of these migrating women had their first HIV consultation in our centre within the first two years of arrival in our country (43% within the first year and 21% within the second year).

At the time of the first HPV screen, median age was 38 years (range: 17–77), median CD4 426 cells/µL (range: 2–1733), prior AIDS had been diagnosed in 104 women (16%), median CD4 nadir was 221 cells/µL and median duration of HIV follow-up was 65.5 months (range: 0–310); 515 (79%) women were on cART (median duration 23 months) with 59% (*n*=366) having undetectable viral load below 50 copies/ml (range:<50–555,000).

The median number of HPV tests was 2 per woman (range: 1–11) with a median interval of 15.9 months (IQ25–75:9–28) between two consecutive tests. HR HPV screening had been performed once in 304 women, twice in 155 women and three or more times in 193 women.

### HR HPV prevalence

The prevalence of HR HPV infection at the first screen was 42.8% (95% CI: 38.9–46.7%) (Table 1). It decreased significantly with age: 65% (below 30 years, *n*=127), 46% (30–39 years, *n*=239), 29% (40–49 years, *n*=201) and 32% (over 49 years, *n*=85) (*p*<0.0001, Chi-square test). The prevalence of HR HPV infection also decreased significantly at higher CD4 cell counts: 63% (below 200/µL, *n*=67), 53% (200– 349/µL, *n*=148), 43% (350– 499/µL, *n*=181) and 28% (over 499/µL, *n*=226) (*p*<0.0001, Chi-square test). The highest prevalence (100%) was found in the youngest women with CD4 count<200 cells/µL and the lowest (between 19 and 22%) in women over 40 years and with CD4 count above 500 cells/µL.

**Table 1:** Prevalence of high-risk (HR) HPV at first screen in univariate analysis by Chi-square test (95% confidence interval (CI))

	N	Prevalence of HR HPV at first screen (95% CI)	Univariate analysis
Age (years)			*p*<0.0001
All	652	42.8% (95% CI: 38.9–46.7%)	
<30	127	65%	
30–39	239	46%	
40–49	201	29%	
>49	85	32%	
CD4 cell count/µL			*p*<0.0001
All	622		
<200	67	63%	
200–349	148	53%	
350–499	181	43%	
>500	226	28%	

**Figure 1:**
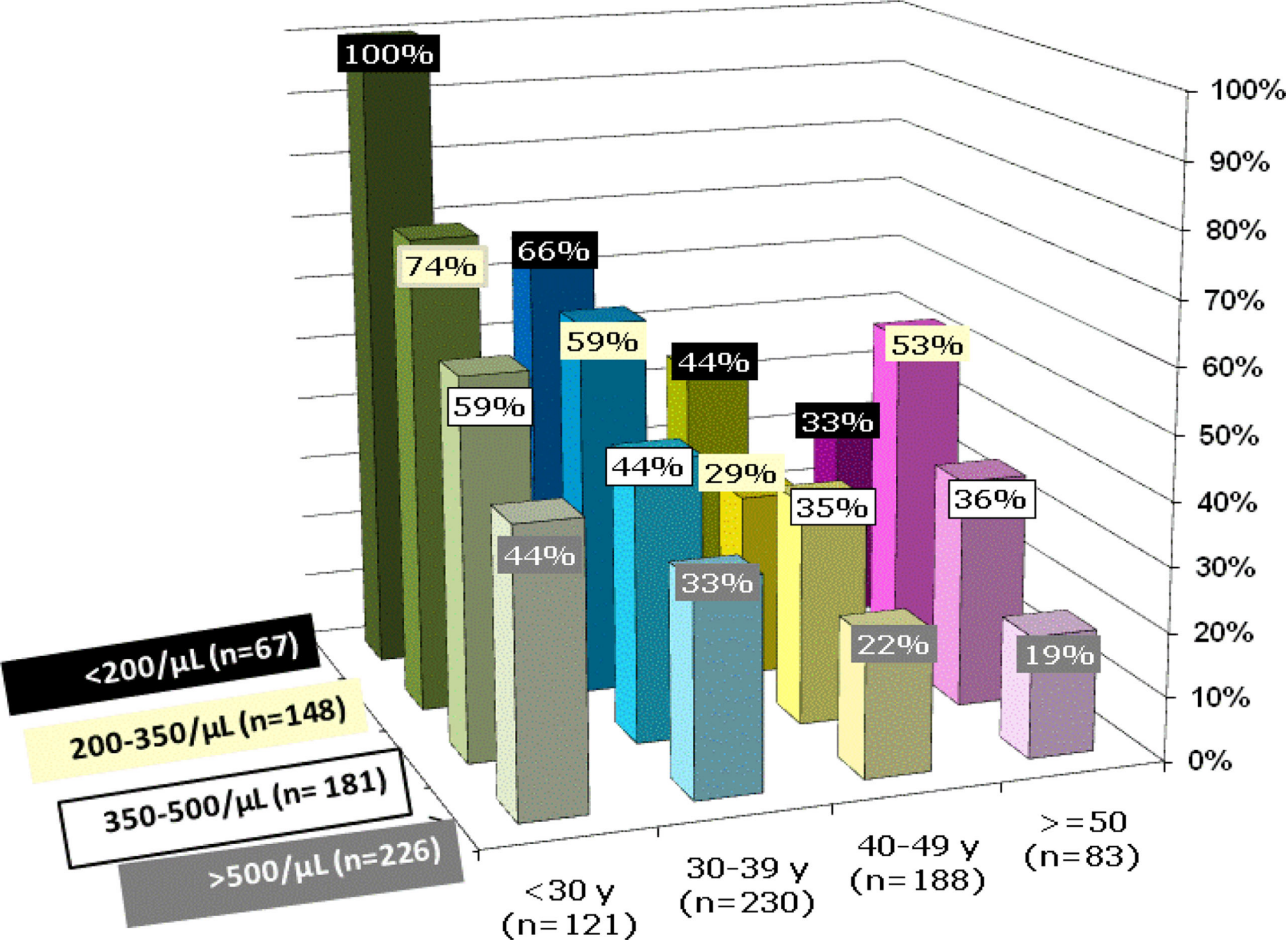
Prevalence of high-risk HPV according to age and CD4 cells count in multivariate analysis. Age in the X-axis (y=years), prevalence in percentage on the Y-axis and CD4 cell count (cells/µL) on the Z-axis; *n*=number of patients included in that group. The prevalence decreases significantly as age increases (*p*<0.0001, logistic regression) and as CD4 cell count raises (*p*<0.0001, logistic regression). The influence of age and CD4 count level were independently statistically significant (*p*=0.06 for interaction, by logistic regression).

The influence of both age and CD4 cell count level on HR HPV prevalence are shown in Figure 1. They were independently statistically significant (*p*<0.0003 and *p*<0.0021, *p*=0.06 for interaction, logistic regression, respectively). We then adjusted for a model free of interaction (*p*<0.0001 for both age and CD4 cell count, logistic regression) and considered age and CD4 cell count as continuous data (Table 2). For each increment of 5 years-age, there was a proportional decrease of the probability to be HR HPV positive at first screen with an odd ratio (OR) of 0.81 (95% CI: 0.74–0.88; *p*<0.0001 by logistic regression) and a RR of 0.89 (95% CI: 0.85–0.93; *p*<0.0001 by Poisson regression); for each increase of 100 CD4 cells/µL, there was also a proportional decrease of the risk of carrying HR HPV with an OR of 0.82 (95% CI: 0.76–0.89; *p*<0.0001) and a RR of 0.89 (95% CI:0.85–.93; *p*<0.0001 by Poisson regression).

**Table 2:** Probability to be high-risk (HR) HPV infected at first screen in multivariate analysis by logistic regression for odds ratio (OR) and Poisson regression for relative risk (RR)

	Probability to be HR HPV at first screen: OR and RR, (95% Confidence Interval) (95% CI)	Multivariate analysis
Age		
for each increment of 5 years	OR=0.81 (95% CI: 0.74–0.88)	*p*<0.0001
	RR=0.89 (95% CI: 0.85–0.93)	*p*<0.0001
CD4+ Lymphocyte cells count/µL		
for each increase of 100 cells	OR=0.82 (95% CI: 0.76–0.89)	*p*<0.0001
	RR=0.89 (95% CI: 0.85–0.93)	*p*<0.0001

HR HPV prevalence was 38.4% among Caucasian women (38/99) and 43.8% for African women (232/530) (*p*=ns, Chi-square test).

Smoking status, previous pregnancies or risk factor for HIV acquisition did not influence HR HPV prevalence. Among the 279 women with HR HPV detected at first screen, the median HIV follow up and cART duration were shorter (47 and 12 months, respectively) than in the 373 HR HPV negative women (77 and 37 months, *p*<0.0001 for both analysis, Chi-square test); these results were statistically significant in univariate but not in multivariate analysis.

### HR HPV incidence

There were 165 patients with a first negative HPV screening followed with at least one subsequent test allowing for the calculation of HR HPV incidence rate. When comparing these 165 women to the rest of the cohort, we found that their median age and CD4 count were higher (40 versus 37 years, *p*=0.0023 and 464 versus 416 cells/µL, *p*=0.008; Wilcoxon test) but that there was no difference in terms of risk factor for HIV acquisition, ethnic origin, smoking status, previous AIDS, CD4 nadir, viral load, length of HIV follow-up or duration of ART. During the 4824 patients-months of follow up, HR HPV was subsequently detected in 54 women leading to an incidence rate of HR HPV of 13.4 per 100 women-years (95% CI: 6.4–20.9). Kaplan-Meier curves for the risk of acquiring new HR HPV infection stratified by age and CD4 cell count level were performed and were not statistically significant (data not shown).

## Discussion

In this prospective observational cohort of HIV-positive women, the prevalence of cervical HR HPV infection is 43%. This is more than three times the prevalence of 12% found in the general population in Belgium and in Europe [11, 12].

As shown previously, we found a significantly higher prevalence in women of younger age and a second peak after 50 years of age [2–4]; nevertheless, prevalence in HIV-positive women remains higher in each age strata than in the general population. HR HPV carriage significantly declines as immune level increases which is also consistent with the literature [3, 4]. However our cohort provides new information on the different prevalence in subgroups of patients according to both age and immunity level underlining the very high prevalence of HR HPV infection in women less than 40 years of age both in the very low CD4 cell count strata as expected but also in the higher CD4 strata. We could also measure that the RR of carrying HR HPV at first screen decreases proportionally by 11% for each increase of 5 years-age and also by 11% for each increment of 100 CD4 cell count/µL.

The prevalence of 43% found in our cohort is higher than those reported in other HIV populations from Western European countries or North-America over the last decade. In a large meta-analysis in HIV-positive women with normal cervical cytology, the HPV prevalence was 31% in North America, 32% in Europe and 57% in Africa [13]. These figures might be overestimating HR HPV prevalence as they reported both LR and HR HPV prevalence together and information on the median age and level of immunity, which are important associated factors, were not available.

In a recent study performed in the United States, the prevalence of HR HPV screened by PCR before initiating cART was 23% among HIV-positive women free of cervical lesions, with a median age similar to our cohort [14]. In a UK study, the prevalence among women with a median age of 33 years and normal cervical cytology was 23% [15]. In another study performed in Spain, the prevalence was 38% in a population with low CD4 cells (269/µL) whereas we found 59% in women of comparable age and immunity level [16].

Our HIV cohort is characterised by a large proportion of patients born in central Africa (Congo, Rwanda and Cameroun) with heterosexual acquisition of HIV and who came to Belgium as adults [17]. The high prevalence of HR HPV that we found is in agreement with other studies performed in Africa. In Zimbabwe, the prevalence of HR HPV was 64% among HIV-positive women with a median age of 25 years, in accordance with our rates of 65% in younger women, although the Zimbabwean cohort included women with abnormal cervical cytology [18]. In a study performed in Rwanda, 42% of HIV-positive women between 35 and 44 years had HR HPV, which is again consistent with our findings [19]. A large proportion of women from our cohort came from countries where age of first sexual intercourses is less than 15 years [20, 21]. As these women came to Europe as adults and were diagnosed HIV-positive shortly after arrival, we may assume that most of them had acquired both HR HPV and HIV before coming to Belgium. However, among women without HR HPV at baseline, the incidence rate of HR HPV new infection was also very high (13.4 per 100 women-year). In contrast, HIV-negative women with similar age and cytology have a much lower incidence of five per 100 women-year [22, 23]. In HIV-positive women, very few studies have generated crude incidence rates for HR HPV and our results are in accordance with a survey performed among younger women (median age 28 years) from Uganda with an incidence of 17 per 100 women-year [24].

Incidence could have been underestimated in our cohort because the median interval between HR HPV screens is 16 months and we could have missed transient new infection or reactivation of HR HPV.

Another potential limitation of our study is the use of Hybrid Capture (HC). HC has been reported to slightly overestimate HR HPV detection when compared to PCR techniques likely due to cross-reaction with LR HPV [25]. However, a Spanish study showed that HC was less sensitive than PCR for screening HR HPV in HIV-positive patients [16].

The higher prevalence and incidence of HR HPV infection found in HIV-positive women might result from different causes: first, direct interaction between HIV proteins and HIV-induced cytokines could favour HPV replication [26, 27]; second, transmission of HIV and HPV share common sexual risk factors such as unprotected intercourse with numerous partners increasing the risk of HPV incident infections; and third, immunodeficiency is associated with less efficient HPV clearance and with higher risk of latent HPV reactivation [3, 28]. This reactivation of HR HPV could explain the higher prevalence and incidence only in a minority of our patients as most of them had previously improved their immune level from 221 to 496 CD4 cells/µL while on cART for a median time of 23 months.

Our findings, as well as other studies, show that HR HPV prevalence and incidence is even higher in HIV-positive women originating from Africa. A recent study in South Africa among heterosexual couples showed that the prevalence of HR HPV infection is 43% in men when both partners are HIV-negative and 70% when they are both HIV-positive [29]. These results suggests that the risk for a woman to be HR HPV infected by a new partner is high in sub-Saharan Africa. In addition, non-European variants of HPV 16 have been found more frequently in African and African American than in Caucasian women [30]. This could reflect sexual mixing behaviours suggesting that people born in Africa are more likely to have an African-born partner even if they are living outside Africa. Having an African-born partner who is himself at higher risk of carrying HR HPV might explain the very high incidence of new HR HPV infection found among our patients.

Increased susceptibility to HPV infection could also be due to immune-genetic differences: some major histocompatibility complex haplotypes have been associated with increased risk of CC according to ethnic origin [31]. Finally, interaction between HIV and HPV might favor and amplify both infections. Recently, HPV infection has been found to be a significant risk factor for HIV acquisition [32]; conversely, as mentioned above, HIV enhances HPV acquisition and persistence. As sub-Saharan Africa holds the highest rates of both HIV and HPV infection worldwide, this might contribute to fuel both HPV and HIV epidemics [33].

In conclusion, we found a very high prevalence of HR HPV of 43% and a very high incidence rate of 13 per 100 women-years in a HIV cohort of women living in Europe and treated with antiretroviral therapy. The prevalence was highest in women with CD4 lymphocytes less than 200 cells/µL and in women below 40 years even with a CD4 count more than 350 cells/µL. The magnitude of HR HPV epidemiology should be a prompt to evaluate the clinical efficacy of vaccination against HPV in HIV-positive women.
